# Cellulose induced protein 1 (Cip1) from *Trichoderma reesei* enhances the enzymatic hydrolysis of pretreated lignocellulose

**DOI:** 10.1186/s12934-021-01625-z

**Published:** 2021-07-19

**Authors:** Hexue Jia, Wan Sun, Xuezhi Li, Jian Zhao

**Affiliations:** 1 State Key Laboratory of Microbial Technology, Shandong University, No. 72, Binhai Road, Qingdao, 266237 Shandong China; 2 National Glycoengineering Research Center, Shandong University, No. 72, Binhai Road, Qingdao, 266237 Shandong China

**Keywords:** Lignocellulose, Cellulase, Enzymatic hydrolysis, Crystal structure, Cellulose induced protein 1

## Abstract

**Background:**

*Trichoderma reesei* is currently the main strain for the commercial production of cellulase. Cellulose induced protein 1 (Cip1) is one of the most abundant proteins in extracellular proteins of *T. reesei*. Reported literatures about Cip1 mainly focused on the regulation of Cip1 and its possible enzyme activities, but the effect of Cip1 on the enzymatic hydrolysis of lignocellulose and possible mechanism have not still been reported.

**Results:**

In this study, Cip1 from *T. reesei* was cloned, expressed and purified, and its effects on enzymatic hydrolysis of several different pretreated lignocellulose were investigated. It was found that Cip1 could promote the enzymatic hydrolysis of pretreated lignocellulose, and the promoting effect was significantly better than that of bovine serum albumin (BSA). And especially for the lignocellulosic substrate with high lignin content such as liquid hot water pretreated corn stover and corncob residue, the promoting effect of Cip1 was even better than that of the commercial cellulase when adding equal amount protein. It was also showed that the metal ions Zn^2+^ and Cu^2+^ influenced the promoting effect on enzymatic hydrolysis. The Cip1 protein had no lyase activity, but it could destroy the crystal structure of cellulose and reduce the non-productive adsorption of cellulase on lignin, which partly interpreted the promoting effect of Cip1 on enzymatic hydrolysis of lignocellulose.

**Conclusion:**

The Cip1 from *T. reesei* could significantly promote the enzymatic hydrolysis of pretreated lignocellulose, and the promotion of Cip1 was even higher than that of commercial cellulase in the enzymatic hydrolysis of the substrates with high lignin content. This study will help us to better optimize cellulase to improve its ability to degrade lignocellulose, thereby reducing the cost of enzymes required for enzymatic hydrolysis.

**Supplementary Information:**

The online version contains supplementary material available at 10.1186/s12934-021-01625-z.

## Background

Lignocellulosic biomass has always been considered a sustainable source for the production of biofuels and chemicals [1]. The bioconversion of lignocellulose into biofuels (such as ethanol) for partial replacing fossil fuels is considered to be a practical way in alleviating energy crisis and environmental protection [2]. During bioconversion of lignocellulose, enzymatic hydrolysis of pretreated lignocellulose to glucose by cellulases is an important step. Different strategies have been reported in literatures to reduce the cost of enzymatic hydrolysis, such as developing effective pretreatment methods [3], screening microorganisms for producing cellulase with high performance [4], engineering modification of enzymes and enzyme systems [4, 5], and improving enzyme recovery efficiency [6], etc. Among them, the development of efficient and cheap cellulase mixture is one of the main research directions of biomass conversion [7].

Cellulase is mixtures composed of complex enzyme systems, which could completely hydrolyze cellulose into glucose by the synergistic action of multiple enzymes. It mainly includes three types of glycoside hydrolases: cellobiohydrolase (CBH), endoglucanase (EG) and β-glucosidase (BG). EG acts on the internal cellulose chain to produce the free chains end. CBH cleaves cellulose chain from the chain ends to form cellobiose, and BG releases glucose by hydrolyzing cellobiose [8]. Moreover, it is also shown that the addition of accessory proteins, such as lytic polysaccharide monooxygenase (LPMO), swollenin, Cip1 and Cip2 (cellulose induced protein 1 and 2, respectively), also play a significant role in the hydrolysis of lignocellulose [9–14]. Adding these accessory proteins is an effective strategy to enhance the efficiency of enzymatic hydrolysis of biomass, thereby reducing the use of cellulase and the saccharification cost of lignocellulose. For example, the addition of LPMO contributes to the rapid liquefaction of biomass under high solid load and reduces cellulase dosage [10]. Addition of swollenin improves the accessibility of cellulase to substrates by promoting amorphization of substrates [11]. Cip2 is capable of cleaving methyl ester of 4-*O*-methyl-d-glucuronic acid, which plays a role in the cleavage of hemicellulose-lignin crosslinking [12].

Cellulose induced protein 1 (Cip1) is one of main proteins in the extracellular secretion of *T. reesei*, with a relative content of about 7% [13]. It was reported that Cip1 consists of a carbohydrate binding module (CBM) and a domain with unknown function [9, 14]. Foreman et al. found that, for various *T. reesei* strains with different cellulase-producing capacities and under different growth conditions, the regulation of Cip1 was indistinguishable from the major cellulases, especially cellobiohydrolase Cel7A [9, 15]. Xia et al. also reported similar results, and found that when the yields of cellulases (cellobiohydrolase CBHI and CBHII, and endoglucanase EGVI) increased, Cip1 was also highly expressed [16]. Jacobson et al. identified the potential enzymatic activity of purified Cip1, and found that there were no hydrolytic activity on the tested plant cell wall components [14]. In addition, the crystal structure of core domain of Cip1 was determined, and structural homology analysis indicated that Cip1 had a high similarity with the lyase [14]. Lehmann et al. used diverse enzyme mixtures for the hydrolysis of pretreated corn stover and identified the proteins variations most correlated to hydrolysis performance on basis of partial least squares regression, and showed that Cip1 was highly correlated with the enzyme performance on pretreated corn stover [17], but this result was derived from the model simulation rather than the experiment, and whether the Cip1 could improve the hydrolysis performance needed further research. Up to now, the studies on Cip1 focused on the regulation of the protein and its possible enzyme activities, and guessing that Cip1 may play a considerable function in the degradation of complex cellulose substrates (catalysis or carbohydrate binding). However, the function of Cip1 in the enzymatic hydrolysis of lignocellulose and the possible mechanism have not been reported. Understanding the role of Cip1 in the hydrolysis of lignocellulose is important for modification of cellulase system to improve the hydrolysis efficiency of cellulase used in the bioethanol industry.

In this study, Cip1 gene of *T. reesei* was cloned and heterologous expressed in a low background strain of *Penicillium oxalicum*. The Cip1 protein was purified and used in enzymatic hydrolysis of several pretreated lignocelluloses for studying the function of the Cip1 in enzymatic hydrolysis of lignocellulose. Possible action mechanism of the Cip1 in enzymatic hydrolysis of lignocellulose was speculated by analyzing the changes in crystallinity of cellulose in pretreated substrates, adsorption of protein into lignin and so on. We think the study will provide very valuable reference for understanding the function of Cip1 protein in enzymatic hydrolysis of lignocellulose, modification of cellulase system for improving hydrolysis efficiency of cellulase, and thus decreasing cellulase cost in bioconversion of lignocellulose.

## Materials and methods

### Materials

Avicel PH-101 was purchased from Aladdin, China. Corncob (CC) and corn stover (CS) provided by Tranlin Group, China were mechanically ground, screened through 10 mesh, collected into plastic bags and mixed for use. Liquid hot water pretreated corn stover (LPCS) and NaOH pretreated corn stover (NPCS) were obtained by cutting the corn stover into 2–3 cm segments, treating with water or 1% NaOH (w/w) at 150 °C for 1 h, respectively, and washing with tap water. Corncob residue (CCR) was the solid residue of corn cob after diluted acid treatment, and provided by Shandong Longlive Biotechnology Inc.

The commercial cellulase preparation SP was a solid enzyme powder produced by Sino Biotechnology Co., Ltd. (Gansu, China), which was fermented by *Penicillium oxalicum* JU-A10 (a resistant mutant strain) [13].

### Protein expression and purification

The Cip1 protein from *T. reesei* (NCBI reference sequence: XP_006961566.1) was expressed in the strain A11Δ with low extracellular protein background (previously constructed in our lab) [18]. Specifically, the gene coding sequence of Cip1 was ligated to a plasmid carrying the promoter of amylase gene *15A* of *Penicillium oxalicum*, the terminator of *trpC* of *Aspergillus nidulans*, and hygromycin B phosphotransferase gene *hph* of *Escherichia coli*. The constructed expression cassette was transformed into protein expression strain A11Δ. The correct recombinant strain was obtained and fermented in 100 ml liquid medium (0.5% glucose, 1.5% starch, Vogel’s) at 30 °C for 3 days, and the extracellular protein was collected by filtration. The HisTrap™ FF column (Histrap, GE Healthcare, USA) was used to purify the recombinant protein Cip1 with C-terminal His6tag. The molecular weight of the recombinant protein Cip1 was determined by SDS-PAGE. Deglycosylation of purified Cip1 was carried out using Endoglycosidase H (Endo H, New England BioLabs, MA) according to the protocol provided by manufacturer. The target band was cut off and analyzed by MALDI-TOF (APT, Shanghai, China). The Cip1-CD (non-CBM region) was also expressed and purified by the above method. The purity of the separated protein is verified by the combination methods of SDS-PAGE and SEC‐HPLC (size exclusion high performance liquid chromatography). The SEC-HPLC was conducted on AKTA avant (GE Healthcare, Madison, WI, USA) using PBS buffer (pH 7.4) as eluent. The flow rate was 0.5 ml/min and the detection wavelength was 280 nm.

### Enzymatic hydrolysis

Enzymatic hydrolysis was carried out with 5% dry mass (DM) in sodium acetate buffer (0.05 M) at pH 4.8, and 1 mg/ml sodium azide was added. The substrate, enzyme and buffer were mixed in 125 ml Erlenmeyer flasks with a reaction volume of 40 ml, and the hydrolysis reaction was conducted in a constant temperature air bath shaker at 48 °C and 150 rpm. The dosage of commercial cellulase SP was 5 mg protein/g DM (approximately 8 FPase units/g DM). In the experiment of adding Cip1 and BSA, the dosage of Cip1 and BSA protein was 0.5 mg protein/g DM. The protein concentration was determined according to the Bradford method [19].

After enzymatic hydrolysis to the specified time, a certain amount of enzymatic hydrolysate was taken and centrifuged at 10,000 rpm for 10 min. The supernatants were obtained by filtration with a 0.22 μm filter. The concentration of monosaccharide was quantitatively analyzed by high-performance liquid chromatography (HPLC) using a Shimadzu refractive index detector (Shimadzu, Kyoto, Japan). The separation was carried out on a Bio-Rad HPX-87P column at 78 °C, using Milli-Q water as eluent. The flow rate was 0.5 ml/min.

The glucan conversion was calculated using the formula (1):

1 $$Glucan\;conversion~\left( \% \right) = \frac{{Glucose\;released\;from\;hydrolysis~\left( {{\text{mg}}} \right)}}{{Substrate\;weight~\left( {{\text{mg}}} \right) \times Glucan\;content~\left( \% \right)}} \times 0.9 \times 100\% .$$

The enhancement coefficient (EC) of additives in enzymatic hydrolysis was calculated by the formula (2):

2 $$EC\left( \% \right) = \frac{{Ca - Cc}}{{Cc}} \times 100\% .$$

Here, Ca and Cc referred to glucan conversions with or without additives, respectively.

### The effect of metal ions and EDTA

In order to evaluate the effect of metal ions and EDTA on the promotion of Cip1, 0.4 M metal ions (Ni^2+^, Mg^2+^, Zn^2+^, Cu^2+^) and EDTA solutions were prepared. Metal ions and EDTA were added into the saccharification system of CCR (with or without Cip1), and the final concentration of metal ions and EDTA in saccharification system was 4 mM. After 72 h of enzymatic hydrolysis, the glucose concentration in the hydrolysate was determined and the glucan conversion was calculated. No metal ions and chemical reagents were added as the control.

### Analysis of potential activity of purified Cip1

Polysaccharide lyase could catalyze depolymerization reaction through β-elimination, forming Δ-4,5-unsaturated ends, resulting in the increase of absorbance at 235 nm [20–22]. The lyase activity of Cip1 was determined by measuring the absorbance of supernatant at 235 nm after hydrolysis. Supernatants from enzymatic hydrolysis process with and without Cip1 were taken at 24 h, 48 h, 72 h, respectively, and absorbance at 235 nm was measured. Meanwhile, in order to avoid the interference of products with cellulase on absorbance measurement, the absorbance at 235 nm after hydrolysis in buffer with Cip1 alone was also measured (no cellulase addition), and same procedure without Cip1 was as control.

Different *p*-nitrophenyl derivatives (Sigma, St. Louis, USA) such as *p*-Nitrophenyl-β-d-glucopyranoside (*p*NPG), *p*-Nitrophenyl-d-cellobioside (*p*NPC), *p*-Nitrophenyl-β-d-xyloside (*p*NPX) and *p*-Nitrophenyl-l-arabinofuranoside (*p*NPAf), as well as sodium carboxymethylcellulose (Yuanye Bio-Technology Co., Ltd., Shanghai, China), beechwood xylan (Sigma, St. Louis, USA) and glucan (Yuanye Bio-Technology Co., Ltd., Shanghai, China) were used as substrates for measuring potential activities of purified Cip1 according to the methods described in the literatures [23–25]. All the activities measurements were performed in 0.05 M sodium acetate buffer (pH 4.8).

### X-ray diffraction (XRD) analysis

The solid residues were obtained by filtration and washed with 50 ml of distilled water. Then samples were dried and ground for XRD analysis. The analysis was conducted using Bruker D8-Advance instrument (Bruker, Germany), and sample was scanned from 2θ = 8° to 2θ = 80°. The data were analyzed by software MDI Jade 5.0.

### Adsorption of cellulase on lignin

Lignin was extracted from LPCS according to the methods described in the literatures [26]. In short, LPCS was repeatedly treated by cellulase until the residual sugar content was less than 2%, and then cellulase was removed by protease to obtain lignin. Lignin (0.03 g) and cellulase (10 mg/g lignin) were added to a 1 ml of reaction system, and incubated in 50 mM acetate buffer (pH 4.8) at 48 °C for 24 h at a shaking speed of 150 rpm. In the experiment of adding Cip1 and Cip1-CD, the dosages of both Cip1 and Cip1-CD protein were 1 mg/g lignin. After incubation, the sample was centrifuged and supernatant was used for different enzymes activities analysis.

### Analysis method

Chemical components of lignocellulosic materials, for example, the contents of cellulose, xylan and lignin, were determined according to the method provided by the NREL method [27]. Filter paper activity (FPA) and the activities of CBH, EG and BG was determined according to the methods in the literature [23–25]. In short, FPA was determined with 50 mg filter paper in 1.5 ml of acetate buffer (pH 4.8, 0.05 M) as substrate, and the reaction was conducted at 50 °C for 1 h. In addition, 1% *p*NPC (with d-gluconic acid-d-lactone as inhibitor), sodium carboxymethylcellulose and *p*NPG were used as substrates to determine the activities of CBH, EG and BG respectively. The reaction was carried out in 0.05 M acetate buffer (pH 4.8) at 50 °C for 30 min. The amount of enzyme required to produce 1 μmol product per minute was defined as a unit of enzyme activity. Statistical analysis of differences between the control and test samples were conducted with the Student’s T-test using the SPSS program (version 22.0).

## Results and discussion

### Expression and purification of Cip1

**Fig. 1:**
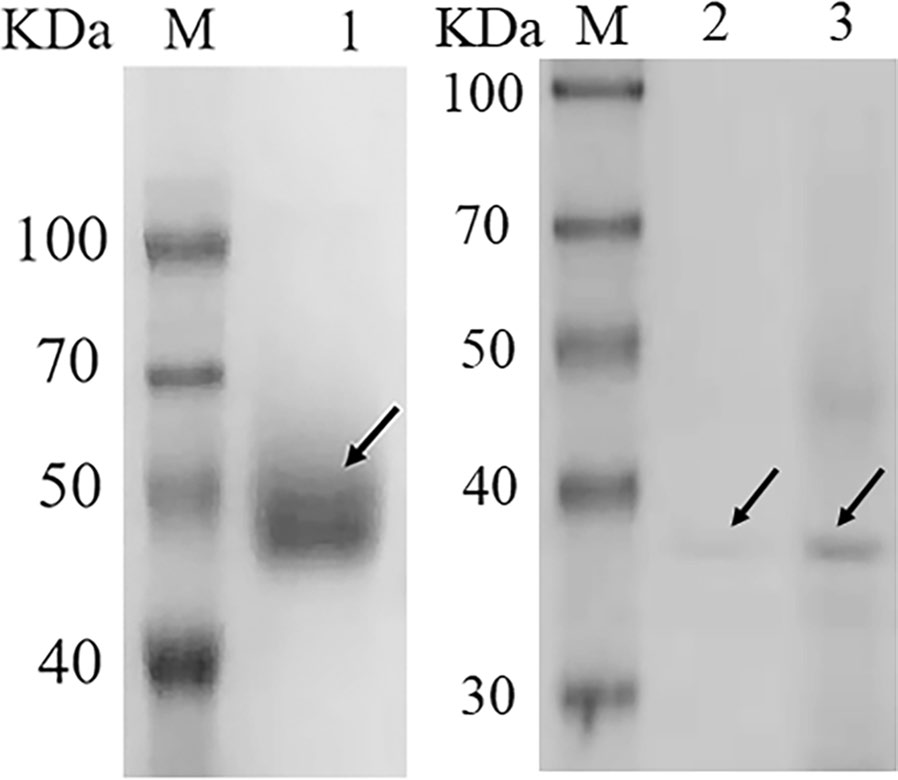
SDS-PAGE analysis of Cip1. Lane M: protein marker; lane 1: purified Cip1; lane 2 and 3: different concentrations of Cip1 after Endo H treatment

In this study, a low extracellular protein background strain A11Δ [18] was used for expressing the Cip1 (NCBI reference sequence: XP_006961566.1) from *T. reesei* QM6a. In brief, the gene coding region of Cip1 was cloned into the vector K-hph-p15A, and then transformed into the strain A11Δ. The recombinant protein Cip1 was produced by using starch as inducer, and purified by HisTrap™ FF column [28]. SDS-PAGE analysis showed that the molecular weight of the purified Cip1 was about 46 KD, as shown in Fig. 1, higher than the calculated molecular weight. The molecular mass of the purified Cip1 decreased after deglycosylation with Endoglycosidase H (Fig. 1), indicating that the increase of molecular weight of purified Cip1 should be due to glycosylation. The Cip1 protein was further identified by mass spectrometry (data not shown).

### Effect of Cip1 on the enzymatic hydrolysis of lignocellulose

**Fig. 2:**
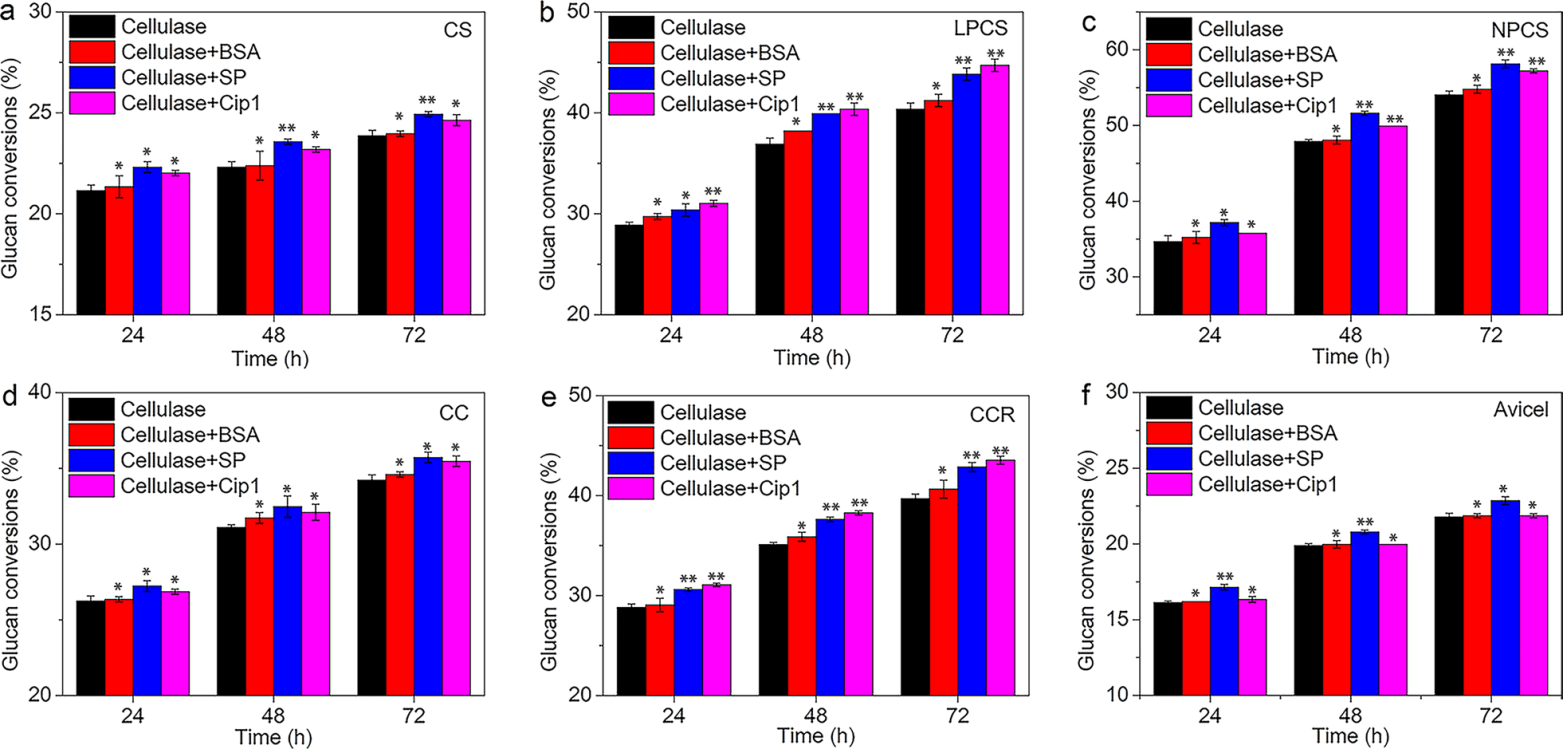
Effect of BSA, commercial cellulase SP and Cip1 addition on enzymatic hydrolysis of CS (**a**), LPCS (**b**), NPCS (**c**), CC (**d**), CCR (**e**) and Avicel (**f**) at 24 h, 48 h and 72 h. In which, protein dosage of 0.5 mg/g DM was added. The data marked with ** represented there were significant difference (p < 0.05) and an asterisk * means that the difference was not significant (p > 0.05)

In this study, various lignocellulosic substrates including corn stover (CS), liquid hot water pretreated corn stover (LPCS), NaOH pretreated corn stover (NPCS), corn cob (CC) and corncob residues (CCR) were selected to assess the effect of the Cip1 protein on enzymatic hydrolysis of lignocellulose. The chemical compositions of the substrates were shown in Table 1. It was found that the contents of hemicellulose and lignin in CS were about 25%. The hemicellulose content of CC was higher than that of CS. As liquid hot water and acid pretreatment removed most of the hemicellulose [26], the hemicellulose contents of LPCS and CCR were only 8.82% and 5.01% respectively. For NPCS, as alkali pretreatment resulted in lignin dissolution [29], lignin content of NPCS was about 9.78%, but more cellulose and hemicellulose components were retained in NPCS. Utilizing these pretreated lignocellulosic materials with different chemical compositions and Avicel as substrates, the effect of Cip1 on the enzymatic hydrolysis was studied by adding Cip1 (0.5 mg/g DM) to commercial cellulase SP (dosage of 5 mg/g DM), and bovine serum albumin (BSA, 0.5 mg/g DM) was substituted for Cip1 protein as control. Figure 2 showed the glucan conversions of enzymatic hydrolysis at 72 h, and enhancement coefficients of the accessory protein on enzymatic hydrolysis with cellulase SP were calculated and shown in Table 2.

**Table 1:** Chemical compositions of different pretreated lignocellulose

Material	Cellulose (%)	Hemicellulose (%)	Lignin (%)	Ash (%)	Extractives (%)
CS	34.79 ± 1.22	25.60 ± 1.20	25.05 ± 0.41	4.03 ± 0.95	10.60 ± 0.51
LPCS	58.06 ± 0.90	8.82 ± 1.90	21.71 ± 0.42	6.95 ± 0.01	4.45 ± 0.35
NPCS	48.30 ± 0.61	22.82 ± 1.64	9.78 ± 0.26	7.35 ± 0.38	9.61 ± 0.90
CC	36.03 ± 0.17	30.89 ± 1.61	18.64 ± 0.22	4.32 ± 0.34	9.87 ± 0.16
CCR	56.68 ± 2.06	5.01 ± 0.28	23.23 ± 0.50	5.22 ± 0.53	7.42 ± 0.28

**Table 2:** Calculated enhancement coefficient of BSA, SP and Cip1 addition on the enzymatic hydrolysis of CS, LPCS, NPCS, CC, CCR and Avicel at 72 h

	CS	LPCS	NPCS	CC	CCR	Avicel
BSA	0.41	2.15	1.38	1.09	2.40	0.41
SP	4.47	8.60	7.59	4.38	8.00	4.96
Cip1	3.25	10.75	5.86	3.65	9.68	0.44

It was found from Fig. 2 that the addition of Cip1 and BSA had little effect on the glucan conversions in the enzymatic hydrolysis of Avicel, but for the enzymatic hydrolysis of lignocelluloses, the glucan conversions were increased after the addition of Cip1. Table 2 showed that enhancement coefficients of Cip1 protein were 3.25%, 10.75%, 5.86%, 3.65% and 9.68% for the enzymatic hydrolysis of CS, LPCS, NPCS, CC and CCR respectively, much higher than that of BSA. And especially for LPCS and CCR, which have high content of lignin in substrates, addition of Cip1 effectively enhanced the enzymatic hydrolysis of lignocellulose. Some studies have shown that addition of BSA could significantly enhance the enzymatic hydrolysis of pretreated lignocellulose with different pretreatment methods [30, 31]. This study showed that the enhancement effect of Cip1 on the enzymatic hydrolysis of lignocellulosic substrates was significantly higher than that of BSA, indicating that Cip1 was a more effective accessory protein for enzymatic hydrolysis of lignocellulose. In addition, it was also found that glucose could not be detected in hydrolysates when only Cip1 was added to the above hydrolysis process (results not shown), which further indicated that the increase in glucan conversions by Cip1 addition was because of the promoting effect of Cip1 on hydrolysis with cellulase, rather than the direct hydrolysis of substrate by Cip1.

Moreover, in order to further evaluate the promotion of Cip1 in enzymatic hydrolysis, commercial cellulase SP with the same protein amount (0.5 mg/g DM) was added into hydrolysis system, and the effect of cellulase addition and Cip1 addition on the enzymatic hydrolysis were also compared. The results were also shown in Fig. 2 and Table 2. It was shown that, whether adding Cip1 or cellulase SP, glucan conversions were increased, that is to say, the addition of Cip1 and cellulase SP enhanced enzymatic hydrolysis of lignocellulose. For pretreated substrates with high lignin content, such as LPCS and CCR, the promoting effect of Cip1 addition on enzymatic hydrolysis (enhancement coefficients was 10.75% and 9.68%, respectively) was higher than that of cellulase SP addition (enhancement coefficients was 8.60% and 8.00%, respectively). However, in the enzymatic hydrolysis of pretreated substrates with low lignin content, such as NPCS, the promoting effect of Cip1 addition was weaker than that of cellulase SP. Especially for Avicel, the promoting effect of Cip1 on enzymatic hydrolysis was significantly lower than that of cellulase SP. It should be attributed to the complex cellulase enzyme system of SP (for example, CBH, EG, BG and so on), compared to the purified Cip1 protein, which could effectively degrade Avicel with high crystallinity of cellulose. For the unpretreated lignocellulose such as CC and CS, the promoting effect of Cip1 addition on hydrolysis was slightly weaker than that of cellulase SP addition, but higher than that of BSA. And possible reason was that, except the effect of enzyme system components, compact structure of cell wall of unpretreated raw materials also affected the hydrolysis efficiency of cellulases. All the results indicated that the addition of Cip1 could effectively promote the hydrolysis of pretreated lignocellulosic materials, especially for lignocellulose with high lignin content, such as LPCS and CCR. That is to say, addition of Cip1 could reduce the dosage of cellulase under the same glucose yield, thus decrease the enzyme cost in enzymatic hydrolysis of lignocellulose. On the other hand, by calculating the enhancement coefficient of Cip1 on the enzymatic hydrolysis of LPCS and CCR at 24 h, 48 h and 72 h, and found that it was maintained at about 10%, indicating that the promoting effect of Cip1 was relatively stable in the hydrolysis process.

### Effects of metal ions and EDTA on Cip1 enhancement

The compositions of biomass are complex and usually contain some minerals. Besides, the pretreatment liquid used in the pretreated process (generally prepared with tap water in industry) may also contain a certain amount of metal ions. Many researches indicated that the presence of metal ions could affect the activity of enzymes. Some metal ions increased the activities of enzymes, but some metal ions led to the loss of enzyme activities [32–34].

**Fig. 3:**
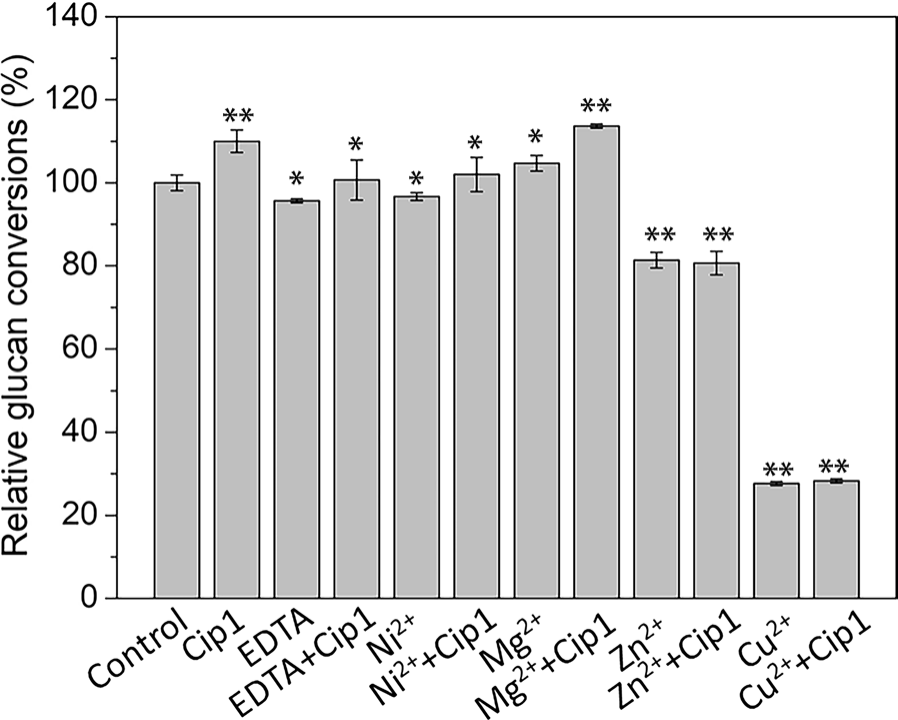
The effect of metal ions and EDTA addition on the promotion of Cip1. No metal ions and chemical reagents were added as the control, and its relative glucan conversion was defined as 100%. The final concentration of metal ions and EDTA was 4 mM in hydrolysis system. The data marked with ** and * respectively means there was significant difference (p < 0.05) and not significant difference (p > 0.05)

In order to verify whether the promoting effect of Cip1 in the enzymatic hydrolysis of lignocellulose was related to metal ions, in this study, Ni^2+^, Mg^2+^, Zn^2+^, Cu^2+^ and EDTA with a final concentration of 4 mM were added to the enzymatic hydrolysis system of CCR, respectively, and the effect of different metal ions and EDTA addition on the promotion of Cip1 were evaluated, as shown in Fig. 3. Compared with the control, addition of EDTA reduced the glucan conversions of CCR, but compared with the addition of EDTA, the simultaneous addition of EDTA and Cip1 still had a promoting effect on the enzymatic hydrolysis. Since EDTA had chelating effect on metal ions, it suggested that the increase of glucan conversions by adding Cip1 was not dependent on metal ions. Figure 3 showed that, compared with the control, the presence of Mg^2+^ in the enzymatic hydrolysis system was beneficial to enhance the hydrolysis efficiency to a certain extent, but not affect the promotion of Cip1 on enzymatic hydrolysis. In general, the addition of Mg^2+^ and Ni^2+^ had little effect on the promoting effect of Cip1. However, Zn^2+^ and Cu^2+^ addition greatly reduced the glucan conversions (18.7% and 72.3% respectively), and also influenced the promoting effect of Cip1 on the enzymatic hydrolysis (compared with adding Zn^2+^ and Cu^2+^alone, the glucan conversion did not increase after adding Cip1 under the presence of Zn^2+^ and Cu^2+^).

Some literatures also reported the effect of metal ions on the enzymatic degradation of lignocellulose. The research from Du et al. showed that the addition of Mg^2+^ could promote the enzymatic hydrolysis of pretreated corn stover [35]. Trace amounts of Zn^2+^ and Cu^2+^ may be necessary for enzyme activities, which acted as enzyme cofactors to produce an activating effect on enzyme and promote enzyme activity [36, 37]. For example, some literature reported that GH61 enzymes are a unique family of copper-dependent oxidases, and the maximum activity of GH61 enzymes required Cu^2+^ [38]. However, excessive Zn^2+^ and Cu^2+^ affected the spatial structure of the cellulase, thereby causing an inhibitory effect on the enzyme activity [39]. Wang et al. also reported that Cu^2+^ and Fe^3+^ inhibited the hydrolysis of cellulose [34]. Our study showed that the presences of Zn^2+^ and Cu^2+^ in enzymatic hydrolysis system seriously affected the hydrolysis efficiency of lignocellulose and promoting effect of Cip1 on the enzymatic hydrolysis, maybe due to the metal ions binding at active sites of cellulases, which inhibited the activities of major cellulases [34].

### Analysis of the potential enzyme activity of Cip1

Through structural homology analysis, Jacobson et al. found that Cip1 had the highest structural similarity with the alginate lyase vAL-1 from *Chlorella virus* and the glucuronan lyase CsGL from *H. jecorina* [14]. The residues at the predicted active site of Cip1 were mostly charged and highly similar to the two lyases [14], suggesting that Cip1 may have lyase activity. The polysaccharide lyase could catalyze the depolymerization reaction through β-elimination to form a product with double bond between C4 and C5 at the non-reducing end. The activity of lyase could be determined by the increase of absorbance at 235 nm owing to the formation of Δ-4,5 unsaturated double bond ends [20–22].

In order to study whether Cip1 had polysaccharide lyase activity, the supernatant from enzymatic hydrolysate of CCR by adding Cip1 was obtained and its absorbance at 235 nm was measured. Table 3 showed that, the absorbance of the supernatant obtained at 24 h, 48 h and 72 h of enzymatic hydrolysis with Cip1 addition was almost similar to the absorbance of supernatant obtained without Cip1 addition. In order to exclude the possible interference of enzymatic hydrolysis products on the absorbance at 235 nm, we only added buffer with or without Cip1 to hydrolysis system of substrate (no addition of cellulase SP), and the absorbances of supernatant from the hydrolysis system at 24 h, 48 h and 72 h were compared. It was also found that their absorbances at same reaction time were similar. These results indicated that Cip1 did not exhibit the activity of polysaccharide lyase, and its promoting effect on hydrolysis should not be attributed to the action of polysaccharide lyase.

**Table 3:** Absorbance of the supernatants at 235 nm during enzymatic hydrolysis of CCR by cellulase or buffer with and without the addition of Cip1 at different hydrolysis time

	Cellulase	Cellulase + Cip1	Buffer	Buffer + Cip1
24 h	0.76 ± 0.004	0.75 ± 0.001	0.49 ± 0.001	0.48 ± 0.002
48 h	0.84 ± 0.001	0.83 ± 0.004	0.49 ± 0.001	0.49 ± 0.002
72 h	0.88 ± 0.004	0.86 ± 0.001	0.50 ± 0.001	0.50 ± 0.002

The supernatants were diluted 20 times before absorbance measurement

Cip1 had a CBM and was co-regulated with the major cellulases from *T. reesei* [15], which suggested that Cip1 may play an important role in lignocellulosic degradation (carbohydrate binding or catalysis). The other possible enzymatic activities related to lignocellulose degradation for the purified Cip1were also analyzed by using *p*NPG, *p*NPC, *p*NPX, *p*NPAf, sodium carboxymethylcellulose, beechwood xylan and glucan as substrates. It was found that the purified Cip1 showed little catalytic activity for the different substrates, which indicated that the promoting effect of Cip1 on enzymatic hydrolysis was not caused by the above-mentioned conventional cellulase and hemicellulase activities.

### XRD analysis of the solid residue after enzymatic hydrolysis

**Fig. 4:**
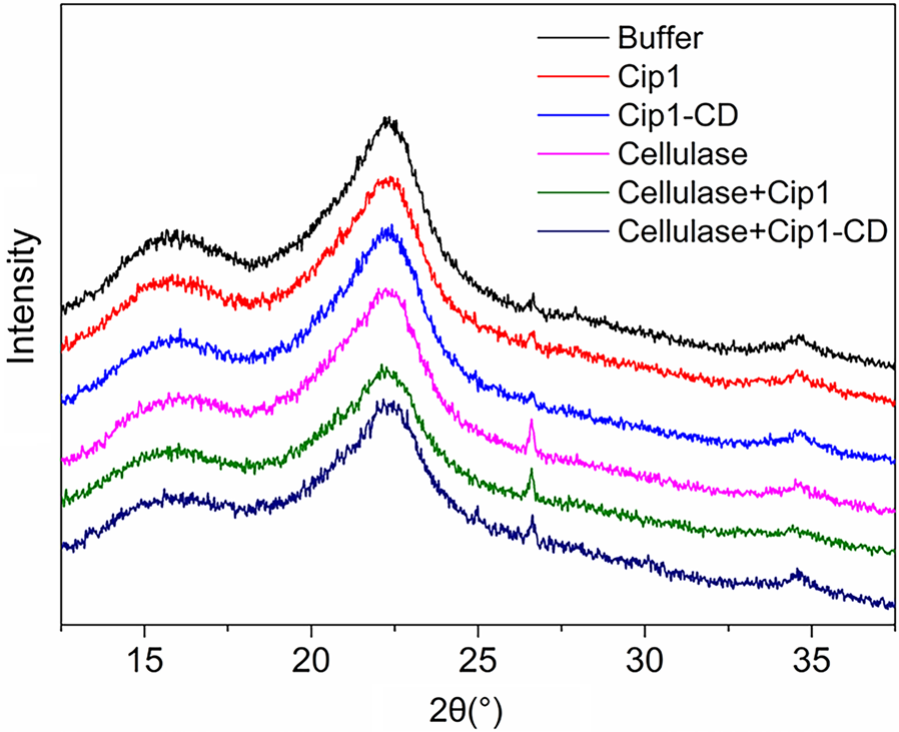
XRD spectra of solid residues after 72 h of enzymatic hydrolysis of CCR by cellulase or buffer with and without the addition of Cip1 and Cip1-CD

The crystalline structure of cellulose is formed by a large number of hydrogen bonds between glucan chains. The crystalline structure reduced the accessibility of cellulase to the substrates and slowed down the saccharification rate, which was an unfavorable factor for enzymatic hydrolysis of lignocellulose [40, 41]. At present, the crystalline structure of cellulose is usually expressed by the crystallinity index (CrI) measured by X-ray diffraction (XRD) [41]. By comparing the X-ray diffraction spectra of the solid residues from enzymatic hydrolysis of CCR by cellulase or buffer with and without the addition of Cip1, the effect of Cip1 on the crystallization of cellulosic substrate was studied. As shown in Fig. 4, after enzymatic hydrolysis for 72 h, the characteristic diffraction peaks of cellulose I appeared at around 16°, 22.5° and 35° [42–44]. Compared with the substrate treated with cellulase alone, the addition of Cip1 significantly decreased the diffraction intensity of the residue at 22.5°. Table 4 also showed that Cip1 addition resulted in the decrease of relative crystallization index of the solid residue after 72 h of enzymatic hydrolysis (from 37.47 to 33.12%). On the other hand, the X-ray diffraction spectra of the solid residues from the hydrolysis with buffer and Cip1 alone were also measured, and Fig. 4 and Table 4 also showed that the addition of Cip1 in buffer could also reduce the relative crystallization index of the residues (from 41.00 to 40.13%). These results indicated that Cip1 could destroy the crystal structure of cellulose, thereby promoting the enzymatic hydrolysis of substrate by cellulase. In addition, in order to determine whether the non-CBM region could reduce the crystallinity of substrate, Cip1-CD was added in enzymatic hydrolysis of CCR and the changes on crystallization index of the solid residues after enzymatic hydrolysis were measured. Table 4 showed that the addition of Cip1-CD also decreased the crystallization index of residue from enzymatic hydrolysis of CCR by cellulase (from 37.47 to 36.14%). Moreover, in order to exclude the possible interference of other impurity proteins on the effect of Cip1-CD, we verified the purity of Cip1-CD through SDS-PAGE and SEC‐HPLC. Additional file 1: Fig. S1 and Additional file 2: Fig. S2 showed that there was only one band and a single peak in SDS-PAGE and SEC‐HPLC, respectively. The results indicated that the change in crystallinity was caused by Cip1-CD, rather than other impurity proteins.

**Table 4:** Crystallization index of solid residues after 72 h of enzymatic hydrolysis of CCR by cellulase or buffer with and without the addition of Cip1 and Cip1-CD

	Crystallization index (%)
Buffer	41.00
Buffer + Cip1	40.13
Buffer + Cip1-CD	40.86
Cellulase	37.47
Cellulase + Cip1	33.12
Cellulase + Cip1-CD	36.14

### The effect of Cip1 on the adsorption of cellulase on the substrate

**Fig. 5:**
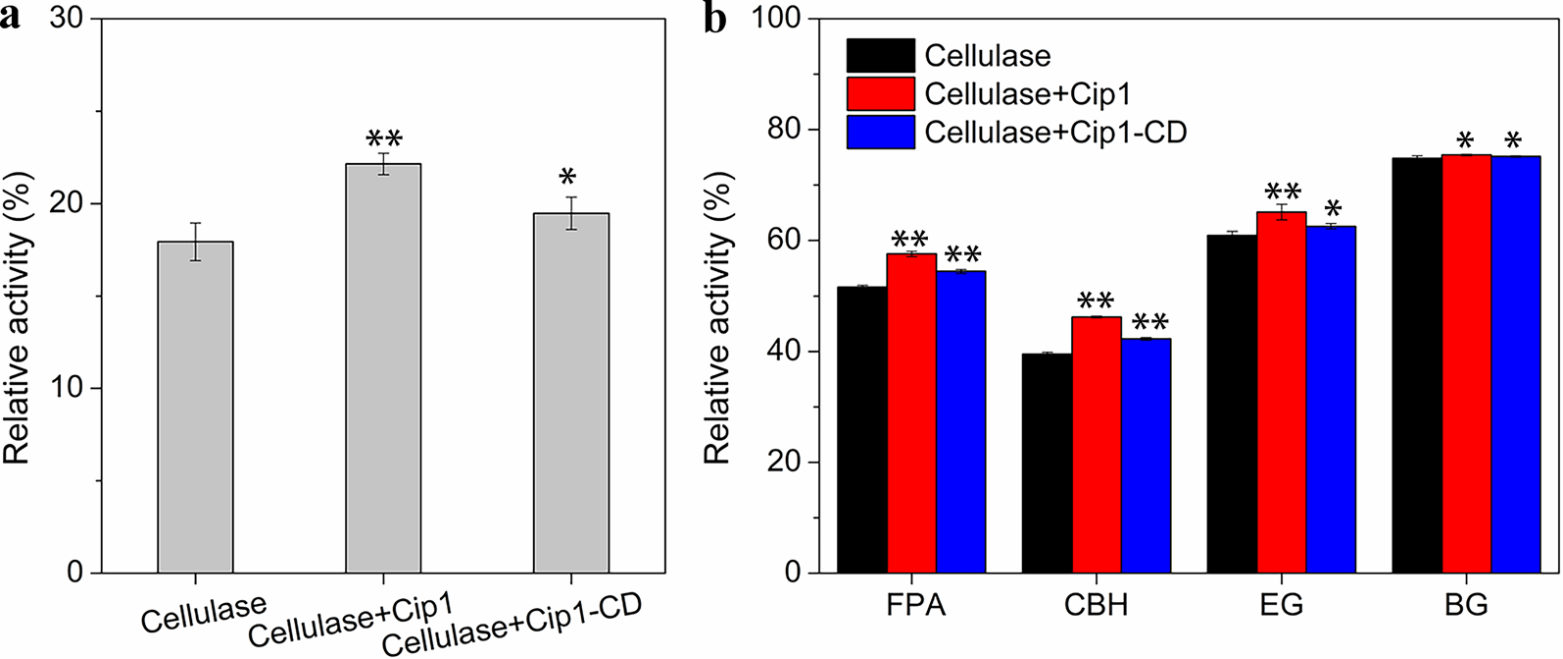
**a** The relative filter paper activity in the supernatant for hydrolysis of CCR at 72 h. **b** The relative activities of FPA, CBH, EG and BG in the supernatant after lignin adsorption for 24 h. The data marked with ** and * respectively means there was significant difference (p < 0.05) and not significant difference (p > 0.05). The relative activity at 0 h of reaction time was defined as 100%

The non-productive binding between lignin and cellulase inhibited cellulase activity and considerably hindered the degradation efficiency of lignocellulose. Lignin-induced non-productive binding has been widely regarded as the main cause of cellulase inactivation and poor enzyme recoverability [45–47]. In this study, CCR was firstly hydrolyzed by cellulase with and without Cip1 for 72 h, and the supernatants of hydrolysate were obtained by centrifugation. Figure 5a showed the relative filter paper activities of the supernatant with the Cip1 addition increased from 17.93 to 22.15%, compared to that without the Cip1, indicating that Cip1 addition increased the activities of free enzymes in the hydrolysis system. On the other hand, using lignin extracted from liquid hot water pretreated corn stover, the effect of Cip1 on different activities of cellulase in supernatant after the adsorption of cellulase onto lignin were further studied. Figure 5b showed that, after adding the Cip1, all the different activities of cellulase in the supernatant were improved, especially CBH (relative activity increased from 39.53 to 46.21%), EG (relative activity increased from 60.93 to 65.13%) and FPA (relative activity increased from 51.59 to 57.58%), while the effect on BG activity was relatively small. These results indicated that the addition of Cip1 could reduce the non-productive adsorption of cellulase protein onto lignin, release more free-cellulase into the supernatant, and thus facilitate improvement of the hydrolysis efficiency of cellulase on substrate. In addition, the effect of Cip1-CD was also studied. Figure 5a, b showed that the addition of only Cip1-CD also slightly increased the relative filter paper activities of the supernatant in hydrolysis system (from 17.93 to 19.47%) and the different enzymatic activities of cellulase in supernatant after the adsorption of cellulase onto lignin. The statistical analysis showed that these changes on enzymatic activities were significant (p < 0.05), indicating that the non-CBM region of Cip1 could also reduce the non-productive adsorption of cellulase protein onto lignin.

**Fig. 6:**
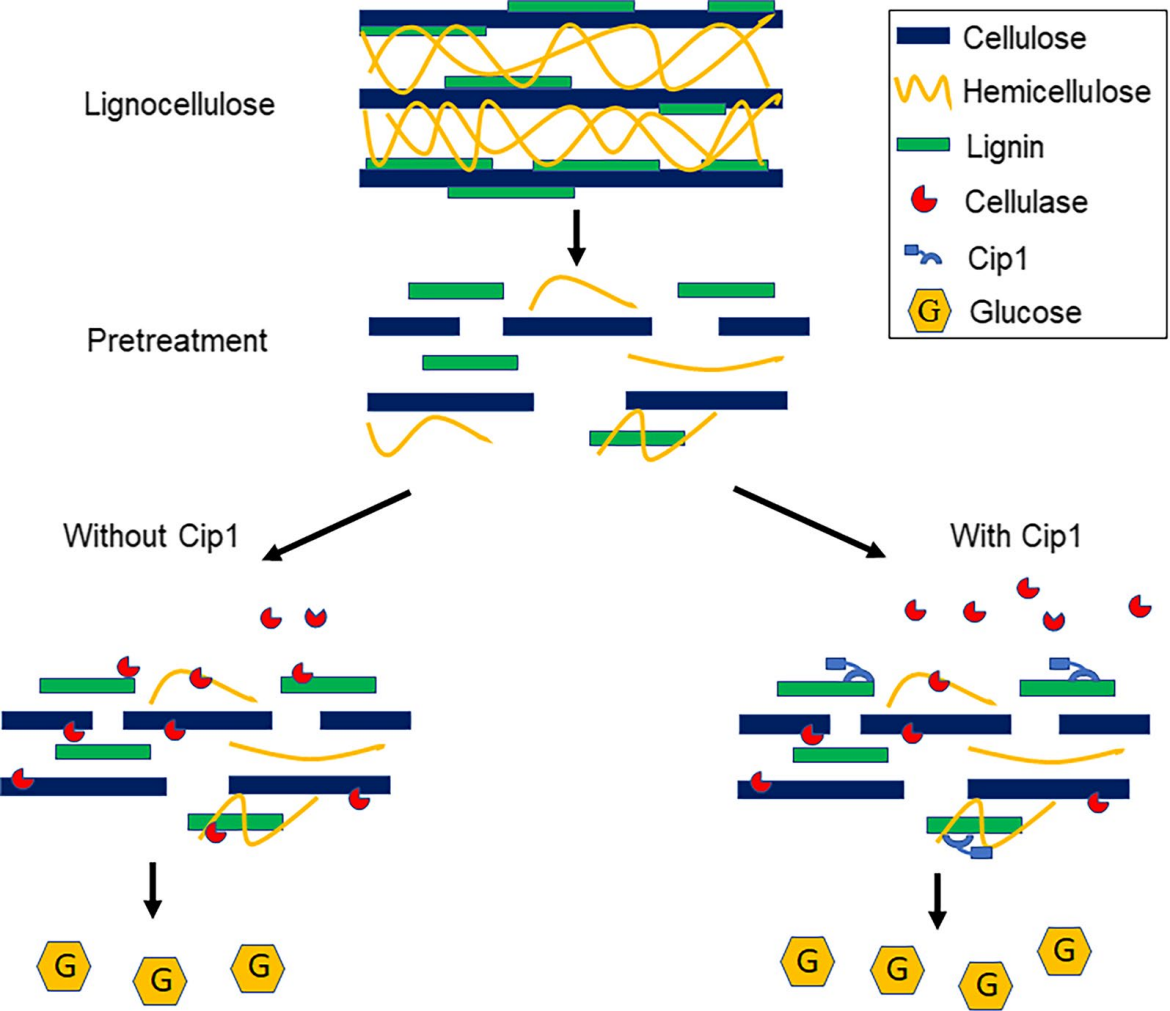
Predicted model of Cip1 enhanced enzymatic hydrolysis of lignocellulose with cellulase

Pretreatment is an important way to improve the accessibility of cellulase and the enzymatic hydrolysis performance of lignocellulose [48]. However, the properties of lignin changed after pretreatment, resulting in its stronger adsorption capacity for cellulase. Studies have shown that the adsorption capacity of cellulase onto lignin of pretreated wood was 2–6 times that of untreated wood [47]. Therefore, reducing the non-productive adsorption of cellulase onto lignin was one of the considerable strategies to improve the degradation efficiency of lignocellulose. Our research results showed that the addition of Cip1 could reduce the non-productive adsorption of cellulase onto lignin (Fig. 5a, b), which may explain why the addition of Cip1 was more effective for the enzymatic hydrolysis of LPCS and CCR with high lignin content. For the binding of cellulase on substrate, carbohydrate binding module (CBM) was considered to be the main binding domain. The CBM of cellulase from *T. reesei* was wedge-folded, in which the flat surface provided the key (aromatic) residues that strongly interacts with lignin [47]. In addition, the non-CBM region of Cip1 could also reduce the non-productive adsorption of cellulase protein onto lignin (Fig. 5a, b). We speculated that Cip1 competitively adsorbed to lignin under the combined action of intrinsic CBM and non-CBM domain, thus reducing the non-productive adsorption of cellulase on lignin, which resulted in the improvement of the degradation efficiency of cellulase (shown in Fig. 6).

## Conclusion

The Cip1 from *T. reesei* could significantly promote the enzymatic hydrolysis of lignocellulose, and the promoting effect was higher than that of BSA under the same protein amount, although the Cip1 protein did not show the activities of polysaccharide lyase and various cellulose-degrading enzymes. Especially for the substrates with high lignin content such as LPCS and CCR, the promotion of Cip1 on enzymatic hydrolysis was even higher than that of commercial cellulase under the same protein amount addition. The promotion of Cip1 may be partly attributed to destroying the crystal structure of cellulose and reducing the non-productive adsorption of cellulase on lignin. This discovery was helpful to guide the modification of strain extracellular secretion system to enhance the ability of cellulase to degrade lignocellulose, and thus reduce the cost of enzymes required for enzymatic hydrolysis.

## Supplementary Information

**Additional file 1: Figure S1.** SDS-PAGE analysis of Cip1-CD. Lane M, protein marker; Lane 1, purified Cip1-CD.

**Additional file 2: Figure S2.** The purity of Cip1-CD was determined through SEC-HPLC.

## Data Availability

All data generated or analyzed during this study are included in this published article.

## Abbreviations

Cip1: Cellulose induced protein 1
BSA: Bovine serum albumin
CBH: Cellobiohydrolase
EG: Endoglucanase
BG: β-Glucosidase
LPMO: Lytic polysaccharide monooxygenase
CBM: Carbohydrate binding module
CC: Corncob
CS: Corn stover
LPCS: Liquid hot water pretreated corn stover
NPCS: NaOH pretreated corn stover
CCR: Corncob residue
HPLC: High-performance liquid chromatography
*p*NPG: *p*-Nitrophenyl-β-d-glucopyranoside
*p*NPC: *p*-Nitrophenyl-d-cellobioside
*p*NPX: *p*-Nitrophenyl-β-d-xyloside
*p*NPAf: *p*-Nitrophenyl-l-arabinofuranoside
FPA: Filter paper activity
XRD: X-ray diffraction
