# Supplementary figures and images for: Cellulose induced protein 1 (Cip1) from Trichoderma reesei enhances the enzymatic hydrolysis of pretreated lignocellulose
Source: Microb Cell Fact. 2021 Jul 19;20:136. doi: 10.1186/s12934-021-01625-z (PMC8287770; doi:10.1186/s12934-021-01625-z)

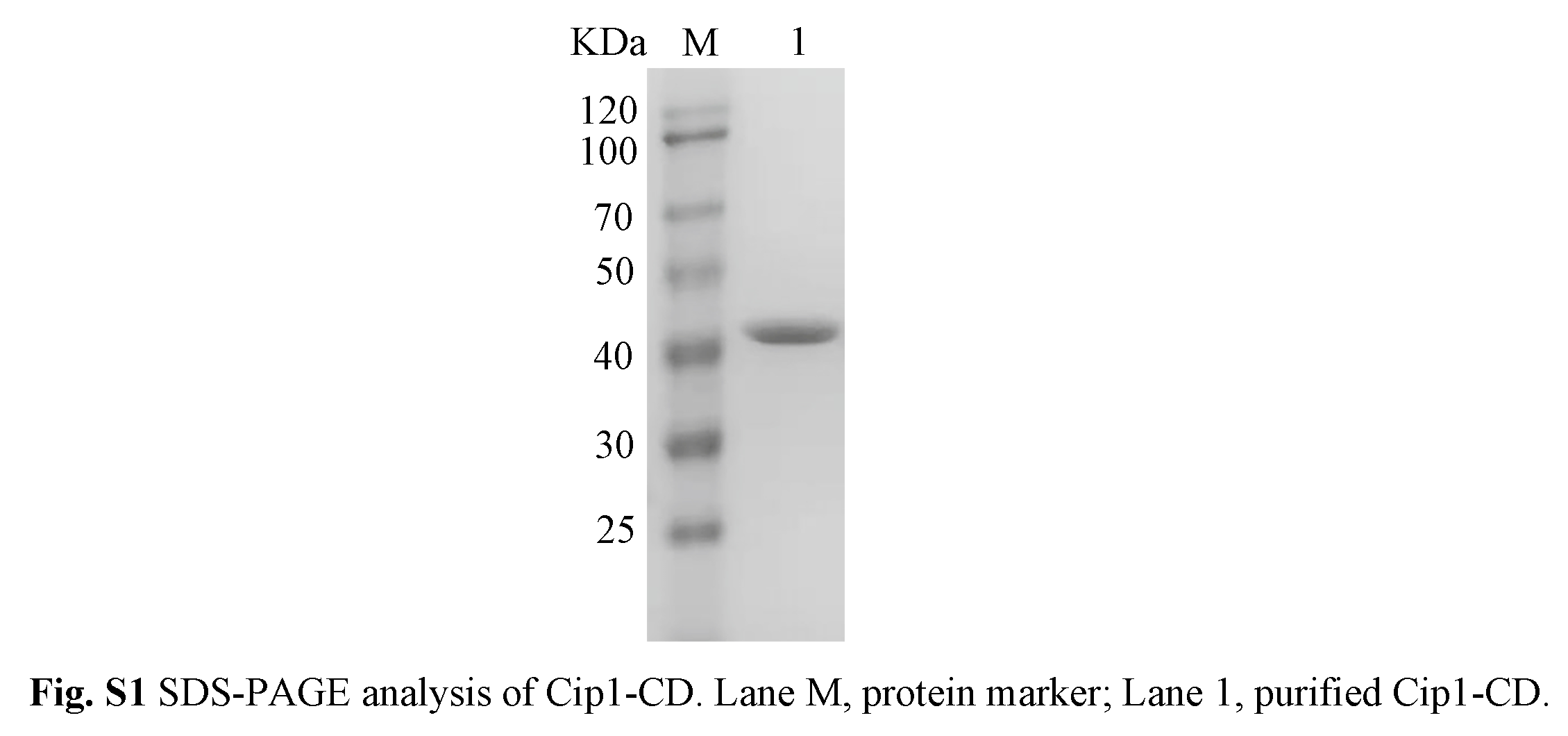

Supplement: Supplementary file 1 — Additional file 1: Figure S1. SDS-PAGE analysis of Cip1-CD. Lane M, protein marker; Lane 1, purified Cip1-CD. [file 12934_2021_1625_MOESM1_ESM.tiff]

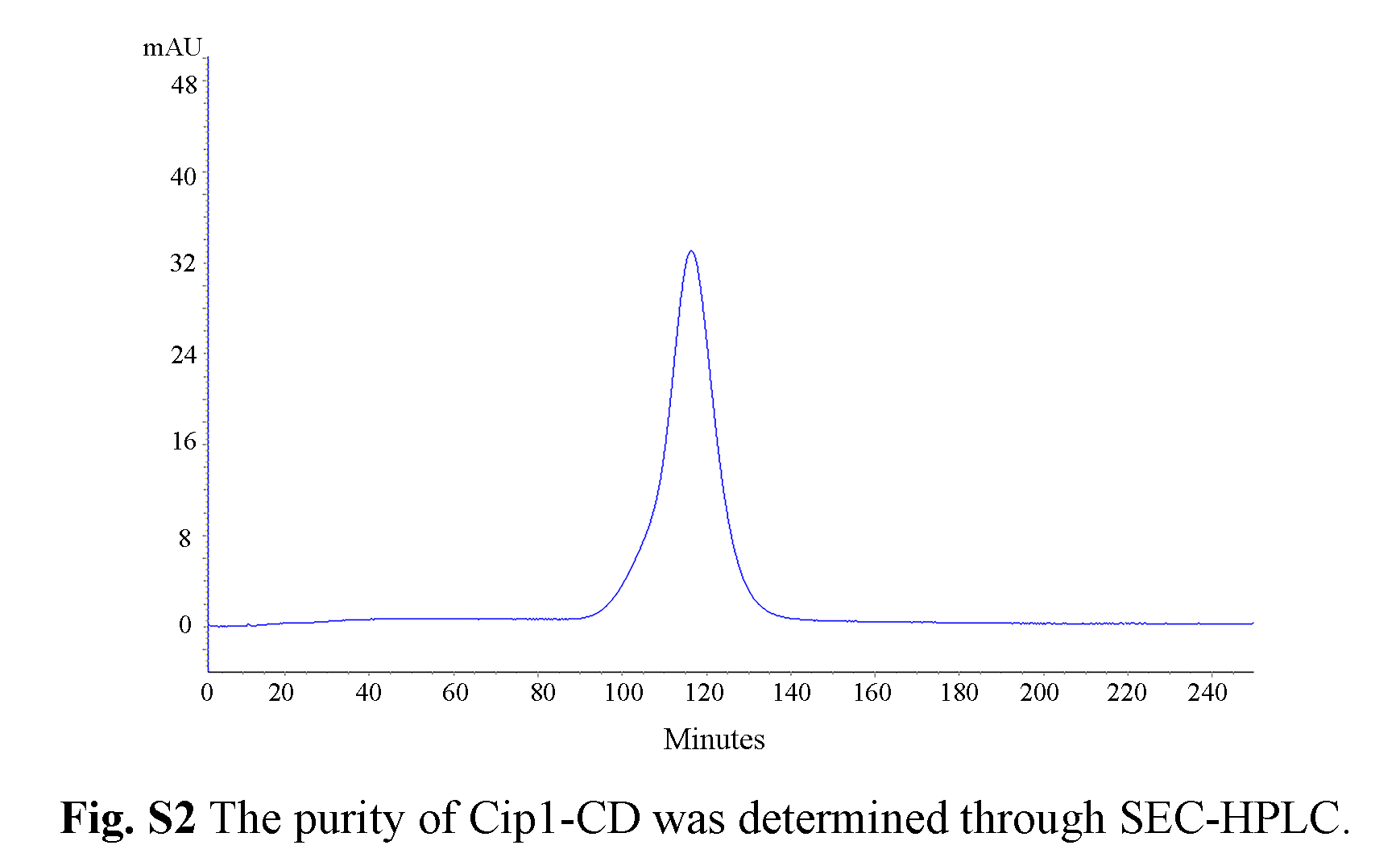

Supplement: Supplementary file 2 — Additional file 2: Figure S2. The purity of Cip1-CD was determined through SEC-HPLC. [file 12934_2021_1625_MOESM2_ESM.tiff]
